# The Brain Activity in Brodmann Area 17: A Potential Bio-Marker to Predict Patient Responses to Antiepileptic Drugs

**DOI:** 10.1371/journal.pone.0139819

**Published:** 2015-10-06

**Authors:** Yida Hu, Xiujuan Mi, Xin Xu, Weidong Fang, Kebin Zeng, Mingming Yang, Chenyu Li, Shasha Wang, Minghui Li, Xuefeng Wang

**Affiliations:** 1 Department of Neurology, The First Affiliated Hospital of Chongqing Medical University, Chongqing, People’s Republic of China; 2 Department of Radiology, The First Affiliated Hospital of Chongqing Medical University, Chongqing, People’s Republic of China; 3 Department of Pediatrics, Chongqing City Hospital of Traditional Chinese Medicine, Chongqing, People’s Republic of China; 4 Department of Neurology, Chongqing City Hospital of Traditional Chinese Medicine, Chongqing, People’s Republic of China; 5 The Nursing Department, Chongqing Three Gorges Central Hospital, Chongqing, People’s Republic of China; 6 The Nursing Department, First Hospital of Shanxi Medical University, Taiyuan, People’s Republic of China; Hangzhou Normal University, CHINA

## Abstract

In this study, we aimed to predict newly diagnosed patient responses to antiepileptic drugs (AEDs) using resting-state functional magnetic resonance imaging tools to explore changes in spontaneous brain activity. We recruited 21 newly diagnosed epileptic patients, 8 drug-resistant (DR) patients, 11 well-healed (WH) patients, and 13 healthy controls. After a 12-month follow-up, 11 newly diagnosed epileptic patients who showed a poor response to AEDs were placed into the seizures uncontrolled (SUC) group, while 10 patients were enrolled in the seizure-controlled (SC) group. By calculating the amplitude of fractional low-frequency fluctuations (fALFF) of blood oxygen level-dependent signals to measure brain activity during rest, we found that the SUC patients showed increased activity in the bilateral occipital lobe, particularly in the cuneus and lingual gyrus compared with the SC group and healthy controls. Interestingly, DR patients also showed increased activity in the identical cuneus and lingual gyrus regions, which comprise Brodmann’s area 17 (BA17), compared with the SUC patients; however, these abnormalities were not observed in SC and WH patients. The receiver operating characteristic (ROC) curves indicated that the fALFF value of BA17 could differentiate SUC patients from SC patients and healthy controls with sufficient sensitivity and specificity prior to the administration of medication. Functional connectivity analysis was subsequently performed to evaluate the difference in connectivity between BA17 and other brain regions in the SUC, SC and control groups. Regions nearby the cuneus and lingual gyrus were found positive connectivity increased changes or positive connectivity changes with BA17 in the SUC patients, while remarkably negative connectivity increased changes or positive connectivity decreased changes were found in the SC patients. Additionally, default mode network (DMN) regions showed negative connectivity increased changes or negative changes with BA17 in the SUC patients. The abnormal increased in BA17 activity may be a key point that plays a substantial role in facilitating seizure onset.

## Introduction

Most neural-psychiatric drugs affect brain activity, but does brain activity affect a patient’s response to drugs? Previous studies have confirmed that changes in brain activity cause varying spontaneous amplitudes of low-frequency (< 0.08 Hz) fluctuations (ALFFs) of the blood oxygen level-dependent (BOLD) signals [1,2]. Using resting-state functional magnetic resonance imaging (fMRI), researchers can assess these changes [3]. Currently, resting-state fMRI studies have been widely used to examine variations in neural activity in many neuropsychiatric disorders, such as temporal lobe epilepsy [4,5,6,7], Alzheimer's disease [8], multiple sclerosis [9,10], schizophrenia [11], and attention deficit hyperactivity disorder [12].

In some cases, the early prediction of a patient’s response to anti-epileptic drugs (AEDs) can lead to a more effective treatment strategy. Drug-resistant (DR) epilepsy is such an example. DR epilepsy is defined as failure to respond to adequate trials of at least two tolerated and appropriately used AED schedules (monotherapy or in combination) to achieve sustained seizure remission [13]. Drug treatment failure can lead to a significant increase in unemployment, divorce, and suicide in these patients [14,15]. Drug-sensitive patients can be treated with AEDs; however, DR patients require more comprehensive therapies, such as surgery, vagus nerve stimulation, immune-mediated therapy and ketogenic diets [16]. It is already known that newly diagnosed epileptic patients who cannot achieve seizure remission when taking the first tolerated AED are more likely to progress to being DR patients [17,18,19]. We termed those newly diagnosed epileptic patients seizure-uncontrolled (SUC) patients, and they are the most important group of patients associated with DR patients who have not yet met the criteria for DR.

The aim of this study was to predict patient response to AED drugs by examining brain activity. We investigated the changes in brain activity in newly diagnosed epileptic patients (seizure-controlled [SC] patients and SUC patients, separately), DR epileptic patients, and well-healed (WH) epileptic patients compared to healthy controls. We hypothesized that common brain activity changes would exist in the DR and SUC patients and that these changes would not be observed in WH and SC patients. We aimed to find a bio-marker with adequate sensitivity and specificity to predict patient responses to AEDs prior to medication.

## Methods

### Participants

This study was performed at the First Affiliated Hospital of Chongqing Medical University. Sixty-nine right-handed subjects participated in this study. All subjects or their guardians provided written informed consent. Recruitment for this study occurred from May 2010 to December 2010. The final follow-up visit was between July 2011 and February 2012. This study was approved by the Committee of Chongqing Medical University and was registered in the Chinese Clinical Trial Register. The clinical registration number is ChiCTR-OCC–14004161.

All study participants met both common inclusion/exclusion criteria and respective inclusion criteria. The common inclusion criteria were as follows: (1) patients with a definite diagnosis of cryptogenic epilepsy with partial secondary generalized seizures. The epilepsy and seizure types were defined according to the International League Against Epilepsy (ILAE) [20]; (2) patients’ willing participation in the study. The common exclusion criteria were as follows: (1) patients with focal abnormalities, as shown by structural magnetic resonance imaging (MRI); (2) patients with progressive central neurological diseases or tumors; (3) patients with only acute symptomatic or non-epileptic seizures; (4) patients with a psychiatric history or mood disorders; (5) patients with epileptic syndromes; (6) patients with neurological deficiencies, such as hearing or visual loss; (7) patients suffering from intolerable adverse events on all selected AEDs during follow-up; (8) patients who were unavailable for follow-up; (9) patients who feared confined spaces; and (10) patients with head motions larger than 2.5 mm of translation or 2.5° of rotation when scanning. The idiosyncratic inclusion for newly diagnosed epileptic patients was patients who had never received AEDs. The idiosyncratic inclusions for DR epileptic patients were patients who matched the standard criteria from the International League Against Epilepsy. The idiosyncratic inclusion criteria for WH patients included: (1) patients without seizure for at least 5 continuous years, and (2) no evident epileptic wave in the most recent Holter monitoring electroencephalogram scan. Thirteen right-handed age- and gender-matched healthy control subjects participated in this study. None had histories of neurological or psychiatric disorders.

### Data Acquisitions

MRI data were collected using a 3.0 T scanner (GE Signa Hdxt) with a standard eight-channel head coil. Headphones and foam pads were used to limit head motion and to reduce noise. Participants were instructed to rest quietly with their eyes closed. Patients were awake during scanning. Functional data were collected using an echo-planar imaging sequence (EPI) with the following parameters: repetition time = 2.0 s, echo time = 40 ms; flip angle = 90°; field of view = 240 mm × 240 mm; matrix = 64 × 64; thickness = 4.0 mm, no gap; and voxel size = 3.75 × 3.75 × 4 mm3. The entire brain was covered in 33 slices, and each functional run contained 240 image volumes.

### Follow-up

Treatment for the newly diagnosed epileptic patients began following MRI scanning. Patient follow-ups occurred at the first, third, sixth, and twelfth months from the initiation of medication. Patients were instructed to record the occurrence of seizures, types of seizures, simultaneous phenomena, adverse events, and hospital admissions in a medical diary. During each follow-up visit, clinical information was recorded based on this information. Patients underwent single drug therapy, as is the recommended practice [21]. Valproate or topiramate monotherapy was the initial medication administered to the majority of patients because, based on our previous clinical studies, these two AEDs are effective and are associated with fewer adverse events for Chinese patients [22,23]. If the seizures were not controlled or the side effects were intolerable with the first AED, treatment was replaced by another AED monotherapy such as lamotrigine, oxcarbazepine or levetiracetam. Table A in S1 File summarizes the information on the medications and seizure types for all of the participants. During the final visit, the physician evaluated the patient response to AED therapy. Patients who had successfully achieved at least 6 months of continuous seizure remission using any tolerable AED therapy were considered to have had a good response to treatment and were consequently placed into the SC group. Patients were placed into SUC group if they had not achieved seizure remission for a minimum of 6 months while receiving any kind of tolerable AED mentioned above.

### Data preprocessing and fALFF calculation

Data preprocessing was conducted using Statistical Parametric Mapping (SPM5, http://www.fil.ion.ucl.ac.uk/spm) and Data Processing Assistant for Resting-State fMRI (DPARSF) [24]. Due to the signal equilibrium and to help participants adapt to the scanning noise, the first 10 volumes of the functional images were discarded. Next, slice-timing correction and motion correction were performed. Patients who had head motions exceeding 2.0 mm or head rotations exceeding 2.0° during the scanning were excluded. Then mean framewise displacement (FD) value of each participant was calculated based on the research from Power and associates [25]. Subsequently, the motion-corrected volumes were spatially normalized to the Montreal Neurological Institute space with a resampling voxel size of 3 mm × 3 mm × 3 mm. Finally, to reduce the effects of low-frequency drifts and high-frequency noise, following smoothing with a 4 mm full-width half-maximum (FWHM) Gaussian kernel, the linear trend and band-pass filtering (0.01–0.08 Hz) were performed on the imaging data.

The Resting-State fMRI Data Analysis Toolkit (REST, http://rest.restfmri.net) [26] was used to calculate the fractional amplitude of low-frequency fluctuation (fALFF). The calculation procedure has been previously described [27,28]. First, the power spectrum was obtained by using a Fast Fourier Transform to convert the time courses into the frequency domain. Second, the square root of the power spectrum was calculated. Third, the square root, averaged across 0.01–0.08 Hz at each voxel, was calculated as the ALFF. Subsequently, the ratio of the power spectrum of the low-frequency range (0.01–0.08 Hz) to that of the entire frequency range was computed as the fractional ALFF (fALFF). Finally, for standardization, the fALFF of each voxel was divided by the global mean of the fALFF value.

### Statistical analyses

#### fALFF statistical analyses

An analysis of variance (ANOVA) was conducted to search for fALFF differences among the SUC, SC, DR, WH, and control groups within the brain mask, and the statistical thresholds were set at P < 0.05 with the minimal cluster size > 4158 mm^3^. Then, two-sample t-tests were performed to determine the fALFF differences for SUC *vs*. controls, SC *vs*. controls, SUC *vs*. SC, DR *vs*. controls, WH *vs*. controls and DR *vs*. WH within the mask based on the ANOVA result, and the statistical thresholds were set at P < 0.05 with the minimal cluster size > 351 mm^3^. The multiple comparisons corrections performed in this study were all computed using the AFNI AlphaSim program (http://afni.nih.gov/afni/docpdf/AlphaSim.pdf) by Monte Carlo simulations [29]. FALFF values of overlaps from SUC vs. CON & DR vs. CON and SUC vs. WH & DR vs. SC were extracted to perform two-sample t-tests using SPSS 13.0 statistical software.

#### Establishing receiver operating characteristic (ROC) curves with fALFF values

Firstly, we extracted fALFF value of the Brodmann 17 (BA17) area for each participant. Then, an ANOVA was conducted to search whether there was BA17 fALFF difference among the SUC, SC, DR, WH, and control groups. Thirdly two-sample t-tests were performed to explore whether BA17 fALFF values could be used to differentiate the SUC patients from controls, SUC from SC patients, DR from controls and DR from WH patients. Finally, ROCs were plotted to identify what cut-point values of fALFF could classify these groups respectively. The statistical thresholds were set at P < 0.05. The ANOVA and two-sample t-tests were performed using SPSS 13.0 statistical software.

#### Functional connectivity (FC) analyses for SUC, SC, and controls

Based on our work, we selected the BA17 area as the region of interest (ROI). The BOLD signal time course was extracted from the ROI. To reduce the effects of high-frequency noise (such as heartbeat and respiration) and low-frequency drift, temporal band-pass filtering (0.01–0.08 Hz) was conducted through a phase-insensitive filter. Following this filtering, the interference, including the effects of head motion parameters, the cerebrospinal fluid region signals, the white matter signals, and global brain signals were eliminated from time series through linear regression[30,31]. Following this adjustment, cross-correlation functional connectivity (FC) analyses were performed by computing the correlations between the ROI time series and the brain voxels time series. Finally, correlation coefficients from each voxel were normalized to Z-scores using Fisher’s r-to-z transformation, and a brain Z-score map was created for each participant.

To create a statistical map that could present significant functional connectivity between the ROI and brain voxels for each group, one-sample t-tests were performed on Z-score maps of SUC, SC and control groups. The significantly positive connectivity, the significantly negative connectivity, and no significant connectivity with the BA17 were defined by one-sample t-test maps. An ANOVA was performed to search FC differences among SUC, SC and healthy controls within brain mask, and the statistical threshold was set at P < 0.05 and was corrected using the AlphaSim criterion with the minimal cluster size > 4158 mm^3^. Two-sample t-tests between each pair of the three groups were then performed to identify FC differences within the mask from ANOVA result, and the statistical thresholds were set at P < 0.05 with the minimal cluster size > 567 mm^3^. The interpretation of the two-sample t-tests results were based on the types of connectivity given by the one-sample t-tests. If the types of connectivity of the two groups were the same based on the one sample t test result, we would use the description of positive connectivity increased change, positive connectivity decreased change, negative connectivity increased change, or negative connectivity decreased change to describe the results. If the types of connectivity of the two groups were different, we would use negative connectivity change or positive connectivity change to point the direction of connectivity changes.

## Results

A total of 69 participants were enrolled in this study, and data from 53 participants (21 newly diagnosed epileptic patients, 8 DR patients, 11 WH patients, and 13 healthy control subjects) were incorporated in the final analysis (S1 Fig). Based on patient follow-up, 11 newly diagnosed epileptic patients were assigned to the SUC group, and 10 patients were enrolled in the SC group. All participants were right-handed. Table 1 illustrates that there were no significant differences among the five groups based on demographic and head motion characteristic.

**Table 1:** Baseline demographic and head motion characteristics for subjects.

Characteristics	SUC	SC	DR	WH	Controls	P value
Number (F/M)	11 (6/5)	10 (4/6)	8 (1/7)	11 (7/4)	13 (8/5)	0.17 ^a^
Scanning age (years) ^b^	29.5±12.8	32.2±17.5	29.5±15.7	29.1±13.7	32.2±11.2	0.97 ^c^
Education years ^b^	12.8±3.4	12.2±3.6	10.6±3.2	11.2±3.9	13.5±5.0	0.47 ^c^
Mean FD (mm) ^d^	0.78±0.25	0.80±0.27	0.85±0.30	0.11±0.13	0.90±0.38	0.73 ^c^

^a^The P values for the gender distributions in the five groups were calculated using the chi-squared test.

^b^The values are represented as mean±standard deviation (SD).

^c^The P values were calculated by one-way ANOVA tests.

^d^The head motion characteristics was represented as mean framewise displacement (FD)±SD.

### fALFF group differences

**Fig 1:**
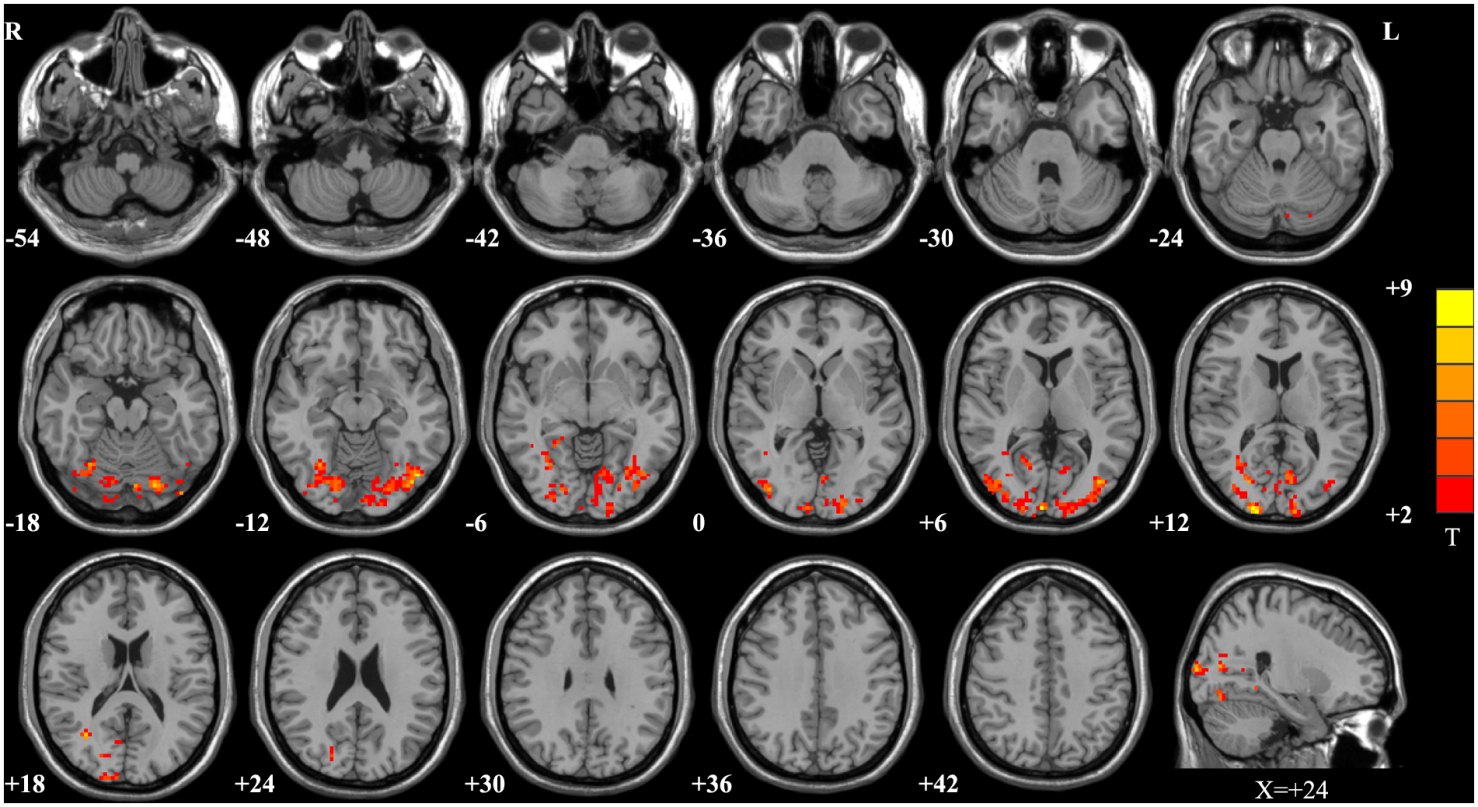
Map of fALFF differences among the SUC, SC, DR, WH and control groups. There were significant fALFF differences among the five groups in the bilateral cuneus, lingual gyrus, inferior/middle occipital gyrus, calcarine, middle temporal-occipital area, fusiform, subcortical structure of left occipital lobe, subcortical structure of right temporal lobe, right posterior cingulated, and right cerebellum posterior lobe. The statistical threshold was set at P < 0.05 and a cluster size > 4158 mm^3^, which corresponded to a corrected P < 0.05.

Fig 1 shows the ANOVA results for the fALFF values among the SUC, SC, DR, WH, and control groups. Significantly different fALFF regions were located in the occipital lobe and temporal lobe, especially in the bilateral cuneus, lingual gyrus, inferior/middle occipital gyrus, calcarine, middle temporal-occipital area, and fusiform. Table 2 shows a detailed list of these regions.

**Table 2:** Regions showing fALFF differences among SUC, SC, DR, WH patients and healthy controls.

Brain region ^a^	BA ^b^	T value	X ^c^	Y	Z
Left cuneus	NA	4.30	-6	-72	12
Right cuneus	NA	4.45	21	-81	21
Left lingual gyrus	NA	5.1	-24	-81	-15
Right lingual gyrus	NA	4.79	18	-78	-12
Right superior occipital gyrus	19	6.86	21	-99	12
Left middle occipital gyrus	19	6.21	-39	-78	6
Right middle occipital gyrus	19	6.09	39	-84	3
Left inferior occipital gyrus	19	7.12	-48	-72	-12
Right inferior occipital gyrus	19	3.38	36	-81	-12
Left calcarine	NA	4.30	-6	-72	12
Right calcarine	NA	3.67	18	-63	6
Left fusiform	19	5.99	-21	-81	-18
Right fusiform	NA	5.15	30	-63	-15
Left middle temporal-occipital area	19	3.02	-39	-75	3
Right middle temporal-occipital area	NA	3.58	51	-75	6
Subcortical structure of left occipital lobe	NA	4.41	-30	-69	-6
Subcortical structure of right temporal lobe	NA	4.69	33	-60	9
Right posterior cingulate	30	3.67	18	-63	6
Right cerebellum posterior lobe	NA	2.80	33	-63	-18

^**a**^Brain region where the peak voxel was located.

^**b**^The Brodmann area where the peak voxel was located

^**c**^The x, y, z, coordinates of the peak voxel in the Montreal Neurological Institute (MNI) space.

**Fig 2:**
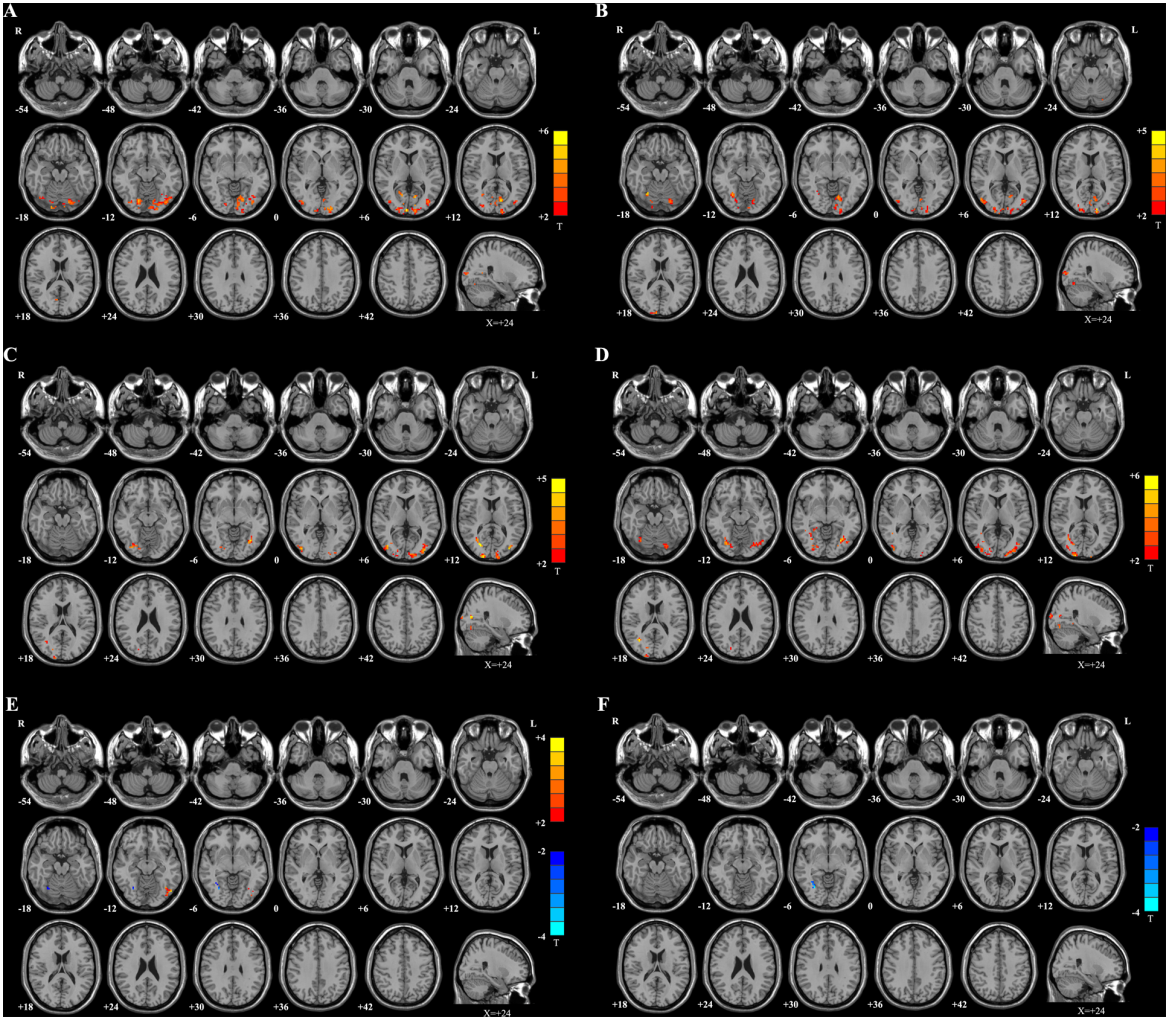
Maps of fALFF differences. **A:** SUC vs. control. Compared with healthy controls, the SUC patients showed significantly increased fALFF values in the warm color regions, including the bilateral cuneus, bilateral lingual gyrus, bilateral superior/middle/inferior occipital gyrus, and right posterior cingulate. **B:** SUC vs. SC. Compared with SC patients, the SUC patients showed significantly increased fALFF values in the warm color regions of the bilateral cuneus, bilateral lingual gyrus, bilateral middle temporal-occipital area, and right fusiform gyrus. **C:** DR vs. controls. The DR patients showed significantly increased fALFF values in the warm color regions of the bilateral cuneus, bilateral middle occipital gyrus, bilateral fusiform, and right middle temporal-occipital area. **D:** DR vs. WH. Compared with WH patients, the DR patients showed significantly increased fALFF values in the warm color regions of the left cuneus, bilateral fusiform, and right middle occipital gyrus. **E:** SC vs. CON. Compared with the healthy controls, the SC patients showed significantly increased fALFF values in the warm color region of the left inferior occipital gyrus. In contrast, the cold color regions in the right fusiform gyrus represent the area with decreased fALFF values in SC patients compared with controls. **F:** WH vs. CON. The WH patients showed only showed decreased fALFF values in the cold color region of the right fusiform gyrus. The statistical threshold was set at P < 0.05 with a cluster size > 351 mm^3^, which corresponded to a corrected P < 0.05.

Fig 2A and Table B in S1 File show the fALFF differences between SUC patients and healthy controls. Only regions with significant fALFF increases were found including the bilateral cuneus, lingual gyrus, superior/middle/inferior occipital gyrus, and right posterior cingulate. Fig 2B shows the comparison of fALFF values between the SUC and SC patients. Interestingly, compared with the SC patients, we also observed significantly higher fALFF values in the bilateral cuneus and lingual gyrus in the SUC patients. The bilateral middle temporal-occipital area and right fusiform gyrus were other parts with higher fALFF values in the SUC patients than the SC patients. Table C in S1 File shows the detailed list of these regions. Fig 2C and Table D in S1 File show the comparison between DR patients and controls. The bilateral cuneus, middle occipital gyrus, fusiform, and the right middle temporal-occipital area had higher fALFF in DR patients than in controls. Fig 2D and Table E in S1 File represent the differences between the DR and WH groups. The bilateral fusiform, the left cuneus and the right middle occipital gyrus showed higher fALFF in DR patients than in WH patients. Fig 2E and Table F in S1 File show two regions have significantly different fALFF values in SC patients compared with healthy controls: the fALFF-increased area of the left inferior occipital gyrus and the fALFF-decreased area of the right fusiform. Fig 2F and Table G in S1 File represent one region with significantly decreased fALFF in the right fusiform in WH patients compared with healthy controls.

**Fig 3:**
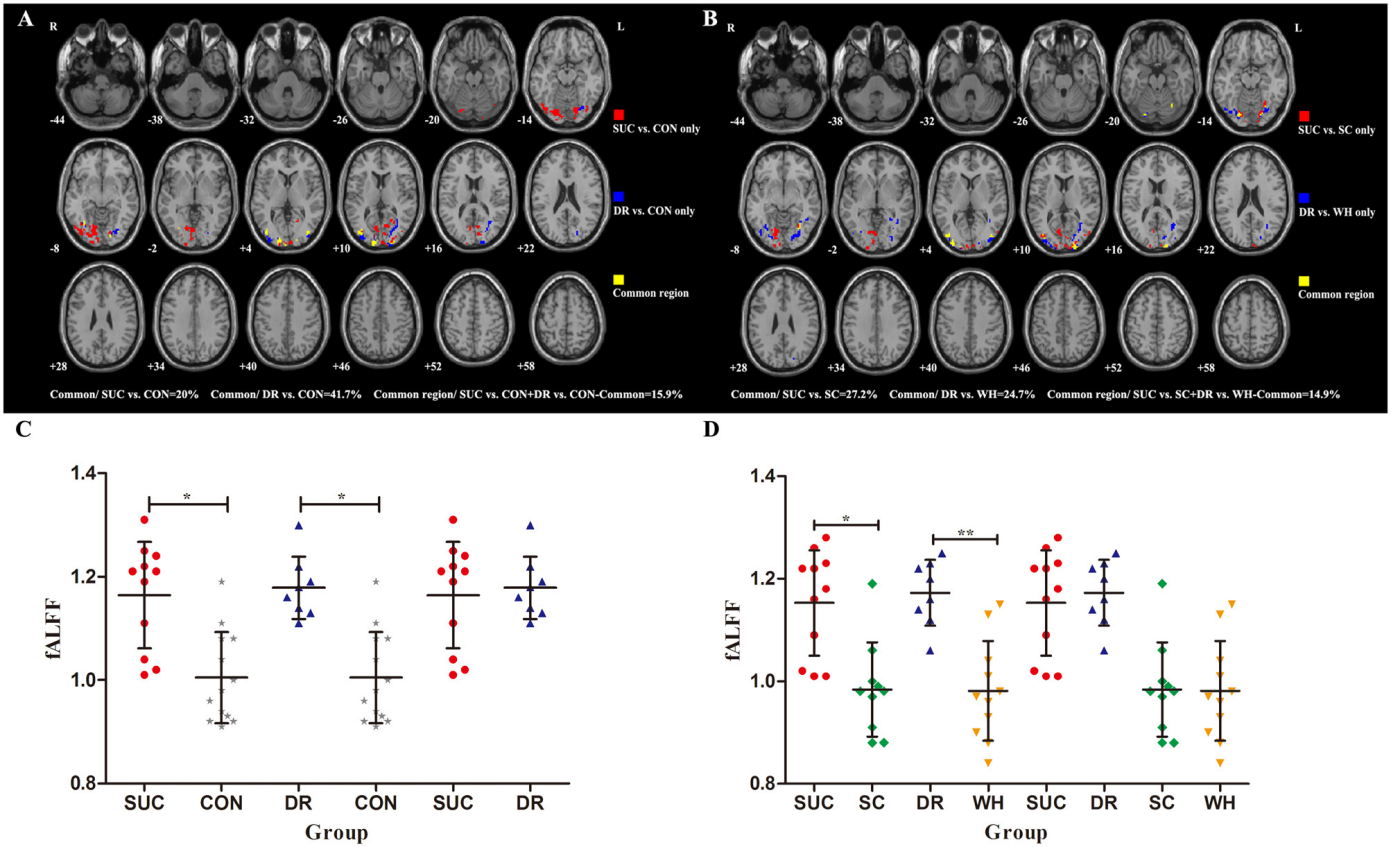
Spatial overlapping maps and scatter plots showing fALFF values of overlaps. **A:** SUC vs. CON and DR vs. CON. Overlap regions account for 20% of SUC *vs*. controls, 41.7% of DR *vs*. controls, and 15.9% of total respectively. The yellow parts represents brain regions with common fALFF changes between SUC *vs*. controls and DR *vs*. controls including the bilateral lingual gyrus, cuneus, inferior occipital gyrus, middle occipital gyrus, right superior occipital gyrus, subcortical structure of left occipital lobe, subcortical structure of right temporal lobe, and left fusiform. The red parts show regions with fALFF differences from comparison of SUC vs. CON only. The blue parts show regions with fALFF differences from comparison of DR vs. CON only. **B:** SUC vs. SC and DR vs. WH. Overlap regions account for 27.2% of SUC *vs*. SC, 24.7% of DR *vs*. WH, and 14.9% of total respectively. The yellow parts represents brain regions with common fALFF changes between SUC *vs*. SC and DR *vs*. WH were found in the bilateral middle occipital gyrus, fusiform, lingual gyrus, the right superior occipital gyrus, cuneus and cerebellum posterior lobe. The red parts show regions with fALFF differences from comparison of SUC vs. SC only. The blue parts show regions with fALFF differences from comparison of DR vs. WH only. All comparisons were restrained in the ANOVA mask. **C:** fALFF values of overlap with common brain activity changes between SUC vs. CON and DR vs. CON. The error bar represents the standard deviation. * P< 0.001. **D:** fALFF values of overlap with common brain activity changes between SUC vs. SC and DR vs. WH. The error bar represents the standard deviation. * P = 0.001, * *P< 0.001.

Spatial overlap regions from SUC *vs*. controls and DR *vs*. controls account for 20% of SUC *vs*. controls, 41.7% of DR *vs*. controls, and 15.9% of total respectively. Fig 3A and Table H in S1 File display the overlap maps. The main common regions were mainly located at the bilateral lingual gyrus, inferior occipital gyrus, middle occipital gyrus and cuneus. 27.2% regions of SUC *vs*. SC, 24.7% regions of DR *vs*. WH and 14.9% regions of both SUC *vs*. SC and DR *vs*. WH was the spatial overlap showing in Fig 3B and Table I in S1 File. The common regions were the bilateral middle occipital gyrus, fusiform, lingual gyrus, the right superior occipital gyrus, cuneus and cerebellum posterior lobe. FALFF values of overlap from SUC vs. controls and DR vs. controls had been extracted to build Fig 3C and no significant difference had been found between SUC and DR patients (P = 0.75). FALFF values of overlap from SUC vs. SC and DR vs. WH had been extracted to build Fig 3D and no significant difference had been found from SUC vs. DR (P = 0.62) or SC vs. WH (P = 0.92).

### ROC analyses

**Fig 4:**
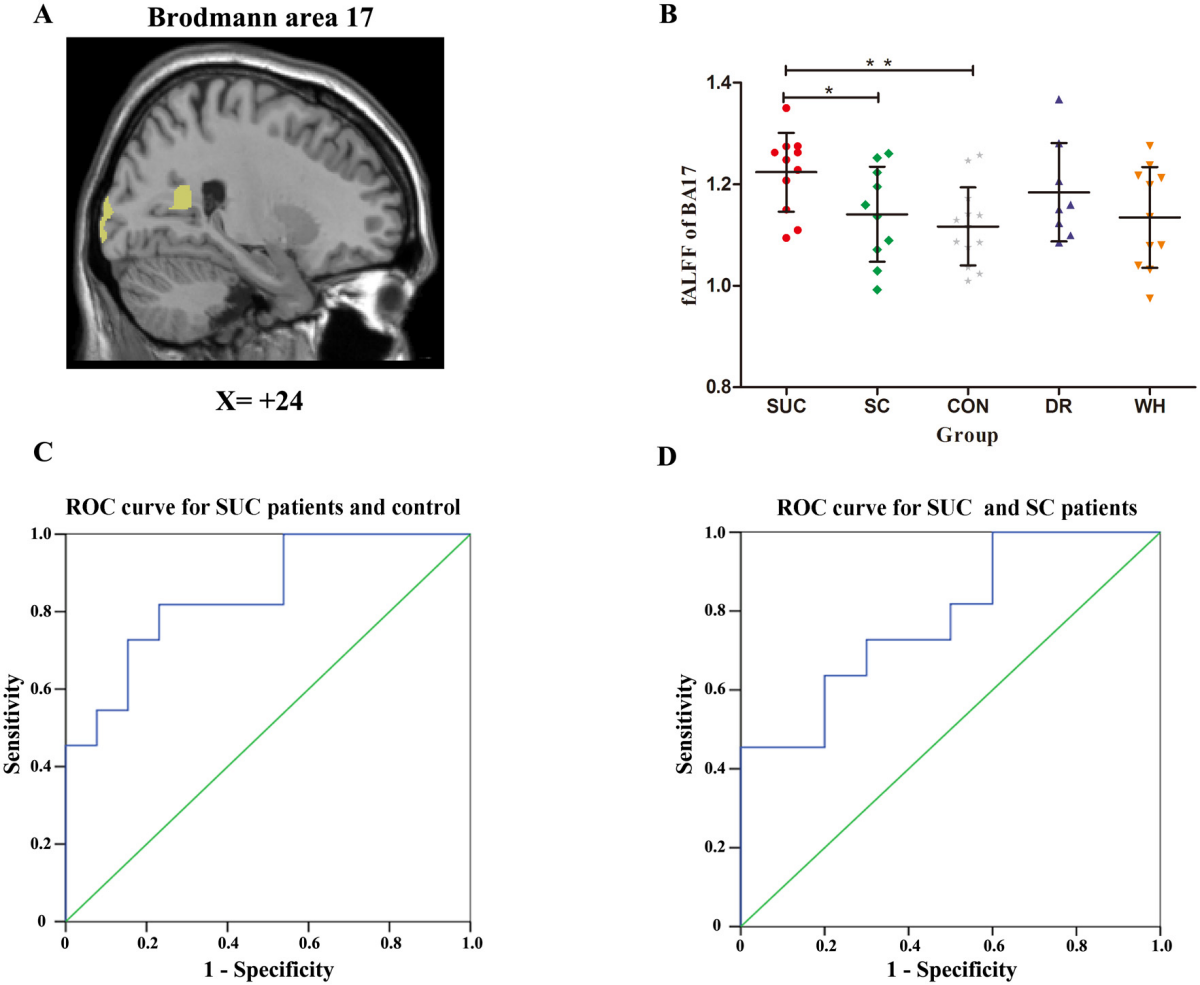
The fALFF values of BA17 and ROC curves. **A:** BA17 mask. **B:** A scatter plot showing fALFF values within BA17 mask in the SUC, SC, DR, WH patients and healthy controls. The error bar represents the standard deviation. * P = 0.01, ** P = 0.026. **C:** An ROC curve for the SUC patients and healthy controls. The cut-off point of the fALFF value for this curve was 1.15. Using this cut-off point, 9 out of 11 SUC patients and 10 out of 13 healthy controls were correctly identified, with a sensitivity of 81.8% and a specificity of 76.9%. **D:** An ROC curve for the SUC and SC patients with a fALFF cut-off point of 1.20. Using this cut-off point, the fALFF of BA17 could classify 8 out of 11 SUC patients and 7 out of 10 SC patients, yielding a sensitivity of 72.7% and a specificity of 70.0%.

Previous results showed both the SUC and DR patients had significantly increased fALFF values in the bilateral cuneus and lingual gyrus while neither the SC nor the WH patients had the same fALFF abnormalities. These findings suggested that the fALFF values for the cuneus and lingual gyrus might serve as markers to detect patients who would have poor responses to AED therapy. Because both the cuneus and the lingual gyrus are located in BA17, we calculated the mean fALFF value of each participant within a BA17 mask (Fig 4A) using the Brodmann area template [32]. ANOVA result indicated there was BA17 fALFF difference among the SUC, SC, DR, WH, and control groups (P = 0.042). Independent two sample t-tests (Fig 4B) showed BA17 fALFF differences were found in SUC vs. CON (P = 0.001) and SUC vs. SC (P = 0.026). No difference was found in DR vs. CON (P = 0.068) and DR vs. WH (P = 0.265). Fig 4C shows the sensitivity and specificity of the fALFF of BA17 for differentiating SUC patients and controls. The area under the ROC curve was 0.85 (P = 0.004, 95% confidence interval [0.690–1.002]). The cut-off point of the fALFF value for this ROC was 1.15. Using this cut-off point, the fALFF of BA17 distinguished 9 out of 11 SUC patients and 10 out of 13 controls, with a sensitivity of 81.8% and a specificity of 76.9%. Notably, the fALFF value of BA17 could also differentiate SUC from SC patients. Fig 4D shows the sensitivity and specificity of the fALFF of BA17 for differentiating SUC and SC patients. The area under the curve was 0.78 (P = 0.029, 95% CI [0.585–0.979]), with a fALFF cut-off point of 1.20. Using this cut-off point, the fALFF of BA17 could classify 8 out of 11 SUC patients and 7 out of 10 SC patients, with a sensitivity of 72.7% and a specificity of 70.0%.

### FC analyses with BA17 as ROI

**Fig 5:**
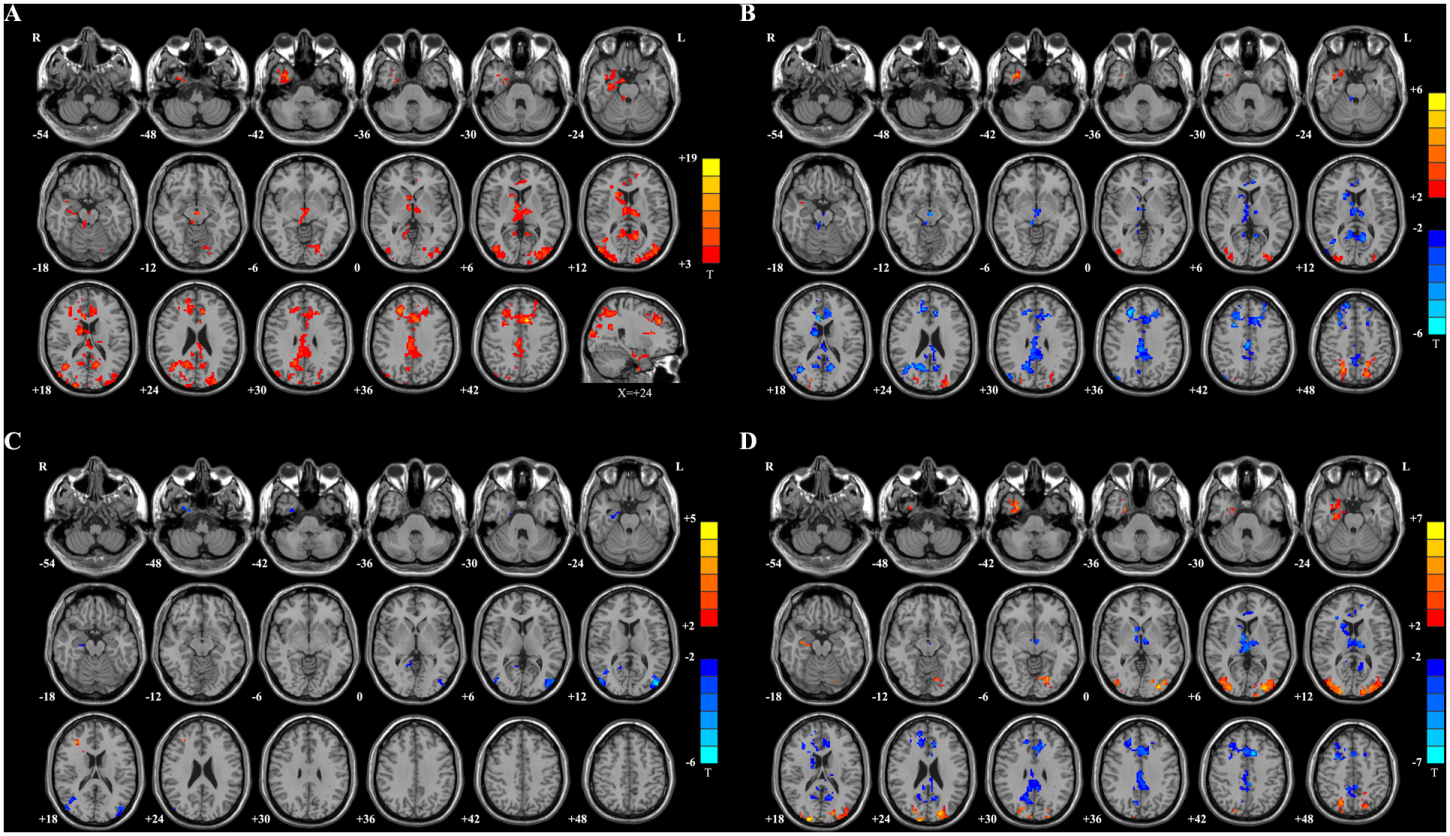
Maps of FC differences. **A:** ANOVA results for SUC, SC and control patients. There were significant FC differences among the three groups in the warm color regions of bilateral precuneus, cingulate, superior/middle/inferior/ frontal gyrus, superior parietal lobule, thalamus, superior/middle/inferior occipital gyrus, fusiform gyrus, middle brain and right parahippocampal gyrus. **B:** SUC *vs*. controls. Compared with healthy controls, the SUC patients showed significantly positive connectivity increased changes or positive connectivity changes in the warm color regions, including the bilateral superior parietal lobule, the bilateral superior/middle occipital gyrus, and the right middle temporal gyrus, while negative connectivity increased changes or negative connectivity changes in the cold color regions, including the bilateral inferior part of precuneus, anterior cingulated gyrus, posterior cigulated gyrus, midbrain, and middle frontal gyrus. **C:** SC *vs*. controls. Compared with healthy controls, the SC patients showed significantly positive connectivity change in the warm color regions located in the subcortical structure of right frontal lobe. The cold color regions included two kinds of connectivity changes: the negative connectivity increased changes or negative connectivity changes were observed in the regions of the right middle temporal gyrus, the right fusiform, and the right parahippocampal gyrus, and the positive connectivity decreased changes were found in the bilateral middle occipital gyrus and the right lingual gyrus. **D:** SUC *vs*. SC. Compared with SC patients, the SUC patients showed significantly positive connectivity increased changes or positive connectivity changes in the warm color regions, including the bilateral superior parietal lobule, superior occipital gyrus, middle occipital gyrus, right fusiform, and right parahippocampa gyrus. Negative connectivity increased changes or negative connectivity changes were observed in the cold color regions of the bilateral inferior part of precuneus, anterior cingulated cortex, posterior cingulate cortex, middle frontal gyrus, and thalamus. The statistical threshold was set at P < 0.05 with a cluster size > 4158 mm^3^ for **Fig 5A** and cluster size > 567 mm^3^ for **Fig 5B–BD**.

One sample t tests results were presented in S2–S4 Figs. Significant regions of FC differences among the SUC, SC and healthy controls were found in the bilateral precuneus, cingulate, superior/middle/inferior/ frontal gyrus, superior parietal lobule, thalamus, superior/middle/inferior occipital gyrus, fusiform gyrus, middle brain and right parahippocampal gyrus (Fig 5A and Table J in S1 File). Compared with the healthy controls, the SUC patients had significantly positive connectivity increased changes or positive connectivity changes in the bilateral superior parietal lobule, the bilateral superior/middle occipital gyrus, and the right middle temporal gyrus and negative connectivity increased changes or negative connectivity changes in the bilateral inferior part of precuneus, anterior cingulated gyrus, posterior cigulated gyrus, midbrain, and middle frontal gyrus. (Fig 5B, Table K in S1 File). Compared with healthy controls, the SC patients showed significantly positive connectivity change in the subcortical structure of right frontal lobe. While negative connectivity increased changes or negative connectivity changes were observed in the regions of the right middle temporal gyrus, the right fusiform, and the right parahippocampal gyrus. The positive connectivity decreased changes were found in the bilateral middle occipital gyrus and the right lingual gyrus (Fig 5C, Table L in S1 File). Compared with SC patients, the SUC patients showed significantly positive connectivity increased changes or positive connectivity changes in the bilateral superior parietal lobule, superior occipital gyrus, middle occipital gyrus, right fusiform, and right parahippocampa gyrus. Negative connectivity increased changes or negative connectivity changes were observed in the bilateral inferior part of precuneus, anterior cingulated cortex, posterior cingulate cortex, middle frontal gyrus, and thalamus (Fig 5D, Table M in S1 File).

## Discussion

We noted several observations in this study. (1) Both SUC patients and DR patients showed significantly increased fALFF changes in BA17, though these abnormalities were not observed in either SC patients or WH patients. (2) The abnormally high level of spontaneous brain activity in BA17 in newly diagnosed epileptic patients significantly correlated with the poor response to antiepileptic drugs. The fALFF value of BA17 could differentiate SUC patients from SC patients and healthy controls with sufficient sensitivity and specificity (3) Regions nearby the cuneus and lingual gyrus were found positive connectivity increased changes or positive connectivity changes with BA17 in the SUC patients, while remarkably negative connectivity increased changes or positive decreased changes in the SC patients. Additionally, default mode network (DMN) regions showed negative connectivity increased changes or negative connectivity change with BA17 in SUC patients. Our findings indicate that the fALFF value of BA17 could serve as a bio-marker to detect patient response to AED therapy prior to treatment.

There are many projections between BA17, the primary visual cortex, sometimes called V1, and other parts of the brain. Once visual signals are analyzed in BA17, they are sent to extrastriate visual areas, such as BA18 (mostly coinciding with V2), BA19 (mostly coinciding with V3), V4, and the middle-temporal (MT) area, for further processing. The signals are then projected to higher visual regions via two major parallel pathways: the dorsal and ventral pathways. The interactive theory of visual consciousness states that the visual signals do not simply travel to the higher areas but that the higher areas send feedback signals back down to lower visual areas, especially to BA17 [33,34]. Even in the resting state, this communication network, with BA17 as the core, still functions [35,36]. Using resting-state fMRI tools, researchers have observed that this visual system has vast information exchanges with other subsystems of brain networks [37]. Altered FC between the visual cortex and other brain regions has been found in many diseases such as Parkinson’s disease [38], blindness [39], cervical dystonia [40], and primary open-angle glaucoma [41]. Studies have confirmed that BA17, when activated, evokes many other parts of the brain [42,43]. Previous research on visually induced seizures has indicated that initial epileptic discharges of BA17 can create dysesthetic symptoms by translating visual signals through the dorsal pathway and can generate limbic lobe symptoms by the ventral pathway [44]. Recently, the existence of drug-resistant epileptogenic networks has been thought to play a substantial role in facilitating seizure onset [45,46,47]. This possibility would indicate that the drug-resistant epileptogenic networks not only prevent AEDs from reaching their target [48] but also disturb brain activity by raising propagations of epileptiform discharges and breaking the balance of neuronal activation and inhibition. In our study, patients with increased brain activity in BA17 in the resting state would possibly have poor seizure control with AEDs. Combining our findings with previous reports, we proposed a hypothesis that the abnormally high level of brain activity in BA17 that was observed in SUC patients may be associated with brain activation and inhibition disturbance. To test this hypothesis, BA17 was selected as the ROI, and functional connectivity analyses were subsequently performed. By examining the low-frequency fluctuations of the BOLD signal of each brain voxel, FC analysis can detect spatial patterns of correlated spontaneous brain activity [2]. It was remarkably different in FC pattern between SUC and SC patients. Taken together, the coherent activity between BA17 and those regions constituted a functional connectivity network, and it is possible that this network has a role in patients’ response to AEDs.

There were notably more brain regions with abnormal spontaneous activity in SUC and DR patients than in SC and WH patients. Interestingly, in SUC and DR patients, all changes were only increased brain activations, in contrast, one brain region showed increased fALFF in SC patients and no region with significantly increased brain activity was found in WH patients. Inter-ictal epileptiform discharges are often detected in the regions with abnormal excitation of neurons by EEG and magnetoencephalography (MEG) [49,50,51]. Previous studies have indicated those regions were closely related with irritative zone and epileptogenic zone, which should be carefully defined in the presurgical evaluation, and could provide valuable information about the epileptogenic network [50,52]. Reports from epileptic surgery suggested accuracy and sufficient extent of resection of the epileptogenic zone could break epileptogenic network and led good postsurgical seizure outcome even seizure freedom [53,54,55]. Our results suggest fALFF value may be considered as a new feature to contribute to presurgical evaluation of epilepsy.

In our study, regions with significantly different FC with the BA17 area were found among the SUC, SC and control groups. Compared with SC patients and healthy controls, SUC patients showed remarkably negative connectivity increased changes or negative connectivity changes in the precuneus, anterior cingulated gyrus, posterior cigulated gyrus, which meant that when increased activity in BA17 was observed in SUC patients, the activity of those regions was more likely inhibited. The regions mentioned above all belong to the default mode network (DMN). The DMN is composed of the posterior cingulated cortex (PCC)/precuneus and the medial prefrontal, anterior cingulated cortex, inferior temporal, and inferior parietal cortices [56]. This network is known to be a “task-negative” network because these brain areas exhibit decreased activity during task-related cognitive processes and increased activity while individuals are in a resting state [1,57,58]. Although the mechanism of DMN formation is unclear, DMN abnormalities have been reported in various neurological and psychiatric disorders [59] such as Parkinson’s disease [60], Alzheimer’s disease [61], epilepsy [6,62], schizophrenia [63], and attention deficit hyperactivity disorder [12]. The abnormalities likely reflect underlying neuronal functional or mental impairments in these patients [59]. Previous studies have suggested that epileptiform discharges or seizures could disrupt function in subcortical regions and could consequently inhibit activity in non-seizing cortical regions [64,65]. The results from recent studies have provided evidence that interictal epileptiform discharges could inhibit the DMN activity [66,67]. It appears to be a recurring pattern whereby the reduced activity of the DMN could facilitate the onset of seizures or epileptiform discharges [68,69,70]. Our results support this viewpoint. It is interesting that SUC patients had notably positive connectivity increased changes in the regions surrounding BA17 area while in SC patients regions near BA17 area had negative connectivity increased or positive connectivity decreased changes. One feature of epileptiform discharges is tending to spread from one region to adjacent structures [71]. If the positive functional connectivity of brain activity between those regions increases, it is easy to understand this change could facilitate the discharge propagation. On the contrary, the regions showing negative connectivity with area BA17 may serve as a fence to prevent epileptiform discharges from spreading out. Nevertheless, the effect of functional connectivity on promoting or inhibiting epileptiform discharge propagation need to identify in further study late. Additionally, only SUC patients had notably positive connectivity with BA17 in the bilateral superior parietal lobule. Previous reports have suggested that the superior parietal lobule have vast neural signal exchanges with the visual cortex in a physiological manner [72,73] Studies have also demonstrated that the abnormal reorganization of physiological networks may be one of the causes of epileptogenesis [74,75,76]. However, whether the there is an epileptic network consisting of BA17 and the bilateral superior parietal lobule in SUC patients and whether the positive connectivity between the two regions would affect patients’ response to AEDs should be carefully investigated in future research.

## Limitations

First, we found that patient AED responses could be predicted by the fALFF values of BA17. However, our results were only based on the fALFF changes in patients with partial secondary generalized seizures. Future studies should investigate whether the present findings are also common in other types of seizures, such as simple partial seizures, complex partial seizures, and the absence of seizures. Second, when abnormal increased fALFF values in BA17 were observed, there were no EEG-fMRI recordings that demonstrated simultaneous epileptic discharges or located their origins. Third, with the purpose of helping physicians forecast potential patient responses to AEDs, we also analyzed factors considered to be associated with prognosis such as age of initial onset, duration of disease, history of febrile convulsions, and number of seizures at baseline [17]. However, no significant differences were detected for these features between SUC and SC patients (Table N in S1 File). One possible reason for this observation may be that our study consisted of a relatively small sample size. However, this result could be interpreted as indicating that the fALFF values in BA17 were more sensitive than previous early identification factors. A larger-scale study would make this theory more persuasive. Fourth, the follow-up period of our study was relatively short; thus, the long-term responses of the patients to AEDs could not be inferred based on only the present results. Fifth, although we removed the linear trend and limited the fMRI data in the band-pass of 0.01–0.08 Hz to reduce the noise from the physiological signals, the respiratory and cardiac fluctuations might remain potential confounders in the final analysis due to the relatively long repletion time (TR = 2.0 s). However, a short TR (e.g., TR = 0.2 s) would make it difficult to image the entire brain. It would be helpful to estimate the effect of physiological shifts by collecting fMRI data and the respiratory and cardiac-linked changes simultaneously in a further investigation. Sixth, to assist subjects in keeping a stable resting state, we asked them to close their eyes during scanning. Therefore, we were unable to compare BA17 fALFF values between the eyes-closed and eyes-open states. Because our results showed that the primary visual cortex played a key role in predicting patient response to AEDs and because some researchers have reported that different resting conditions might lead to different spontaneous brain activity [27,77], it would be interesting to compare the results obtained under the eyes-closed and eyes-open states in further research.

## Conclusion

This study demonstrated that it is possible to predict newly diagnosed epileptic patient responses to AEDs by investigating the fALFF values of spontaneous brain activity using resting-state fMRI scanning. The fALFF value of BA17 is a sensitive and specific marker that can distinguish those patients whose seizures are unlikely to be controlled by AEDs. In those patients who have poor responses to AEDs, BA17 may be associated with an imbalance between brain activation and inhibition. Furthermore, a long-term EEG-fMRI study of a larger cohort, including those with different seizure types, should to be conducted to explore a possible mechanism of human brain activity shifts affecting patient responses to AEDs.

## Supporting Information

S1 Fig Study flow diagram. (TIF)

S2 Fig Map of functional connectivity between BA17 and other voxels in the SUC group. The warm color areas represent the regions with positive connectivity with BA17, including the bilateral postcentral gyrus, the bilateral occipital lobe, the bilateral superior parietal lobule, the bilateral middle temporal gyrus, the bilateral parahippocampal gyrus, and the right fusiform gyrus. In contrast, the cool color areas showed the regions negatively connected with BA17, which were located at the bilateral inferior part of precuneus, the bilateral medial prefrontal cortex, the bilateral superior/middle/inferior frontal gyrus, the bilateral cingulate gyrus, the bilateral thalamus, the bilateral corpus callosum, the bilateral inferior parietal lobule, the bilateral inferior temporal gyrus and the bilateral brain stem. The statistical threshold was set at P < 0.05 and cluster size > 4158 mm^3^, which corresponded to a corrected P < 0.05 (Alphasim-corrected).
(TIF)

S3 Fig Map of functional connectivity between BA17 and other voxels in the SC group. The warm color areas represent the regions that had positive connectivity with BA17, including the bilateral postcentral gyrus, the bilateral inferior part of precuneus, the bilateral posterior cingulate gyrus, the Subcortical structure of right frontal lobe, and the bilateral occipital lobe. In contrast, the cool color areas showed the regions negatively connected with BA17, which were located at the bilateral superior/middle/inferior frontal gyrus, the bilateral anterior cingulate gyrus, the bilateral corpus callosum, the bilateral thalamus, the bilateral supramarginal gyrus, the bilateral superior parietal lobule, the bilateral parahippocampal gyrus, and the bilateral middle/inferior temporal gyrus.
(TIF)

S4 Fig Map of functional connectivity between BA17 and other voxels in the control group. The warm color areas represented the regions that had positive connectivity with BA17, including the bilateral fusiform gyrus, the bilateral inferior part of precuneus, the bilateral posterior cingulate gyrus, the bilateral occipital lobe. In contrast, the cool color areas showed the regions negatively connected with BA17, which were located at the bilateral superior/middle/inferior frontal gyrus, the bilateral anterior cingulate gyrus, the bilateral middle/inferior temporal gyrus, the bilateral superior parietal lobule, the bilateral parahippocampal gyrus, the bilateral corpus callosum, the bilateral supramarginal gyrus, and the bilateral brain stem.
(TIF)

S1 File This file contains Tables A-N. **Table A.** Seizure type and medication information for the patients. **Table B.** Regions showing fALFF differences between SUC patients and healthy controls. **Table C.** Regions showing fALFF differences between SUC patients and SC patients. **Table D.** Regions showing fALFF differences between DR patients and healthy controls. **Table E.** Regions showing fALFF differences between DR patients and WH patients. **Table F.** Regions showing fALFF differences between SC patients and healthy controls. **Table G.** Regions showing fALFF differences between WH patients and healthy controls. **Table H.** Overlap regions with common fALFF changes between SUC vs. CON and DR vs. CON. **Table I.** Overlap regions with common fALFF changes between SUC vs. WH and DR vs. SC. **Table J.** Significant differences in functional connectivity to the BA17 area among SUC, SC and healthy controls. **Table K.** Significant differences in functional connectivity to the BA17 area between SUC patients and healthy controls. **Table L.** Significant differences in functional connectivity to the BA17 area between SC patients and healthy controls. **Table M.** Significant differences in functional connectivity to the BA17 area between SUC and SC patients. **Table N.** Comparison of clinical features associated with early identification of patient’s response to AEDs between SUC and SC group.
(DOC)
