# Supplementary figures and images for: The Brain Activity in Brodmann Area 17: A Potential Bio-Marker to Predict Patient Responses to Antiepileptic Drugs
Source: PLoS One. 2015 Oct 6;10(10):e0139819. doi: 10.1371/journal.pone.0139819 (PMC4595505; doi:10.1371/journal.pone.0139819)

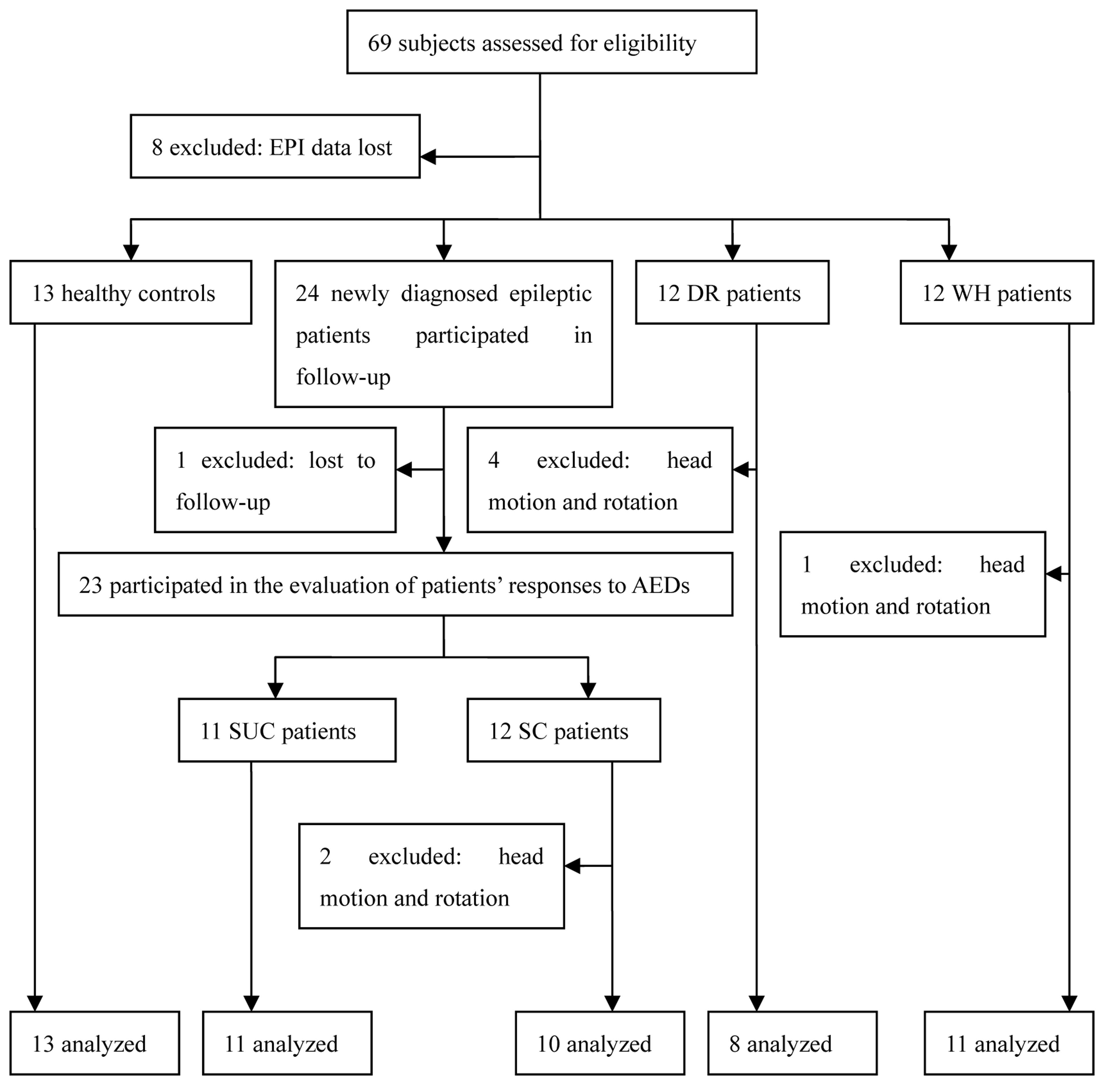

Supplement: S1 Fig — (TIF) [file pone.0139819.s001.tif]

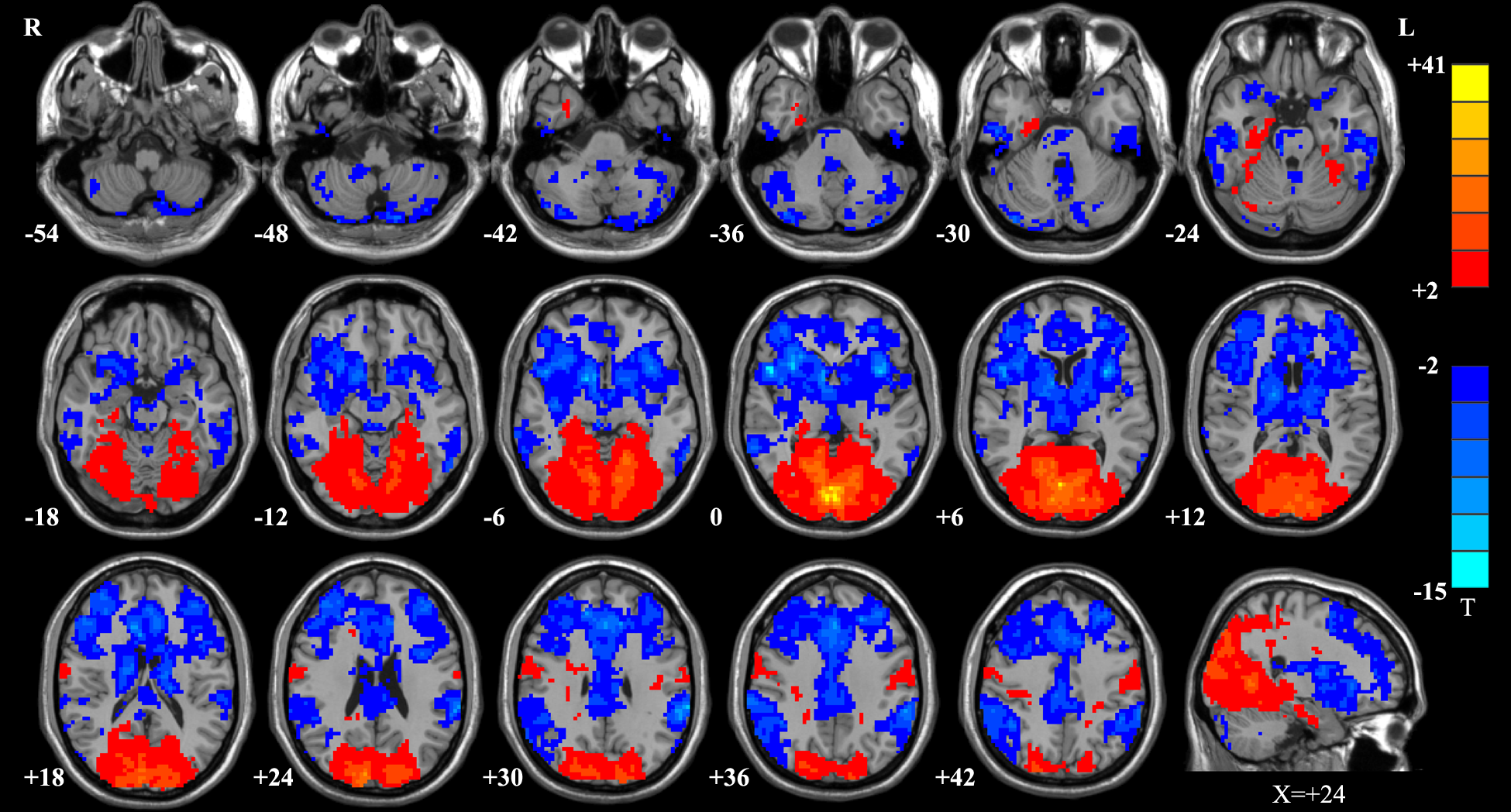

Supplement: S2 Fig — The warm color areas represent the regions with positive connectivity with BA17, including the bilateral postcentral gyrus, the bilateral occipital lobe, the bilateral superior parietal lobule, the bilateral middle temporal gyrus, the bilateral parahippocampal gyrus, and the right fusiform gyrus. In contrast, the cool color areas showed the regions negatively connected with BA17, which were located at the bilateral inferior part of precuneus, the bilateral medial prefrontal cortex, the bilateral superior/middle/inferior frontal gyrus, the bilateral cingulate gyrus, the bilateral thalamus, the bilateral corpus callosum, the bilateral inferior parietal lobule, the bilateral inferior temporal gyrus and the bilateral brain stem. The statistical threshold was set at P < 0.05 and cluster size > 4158 mm3, which corresponded to a corrected P < 0.05 (Alphasim-corrected). (TIF) [file pone.0139819.s002.tif]

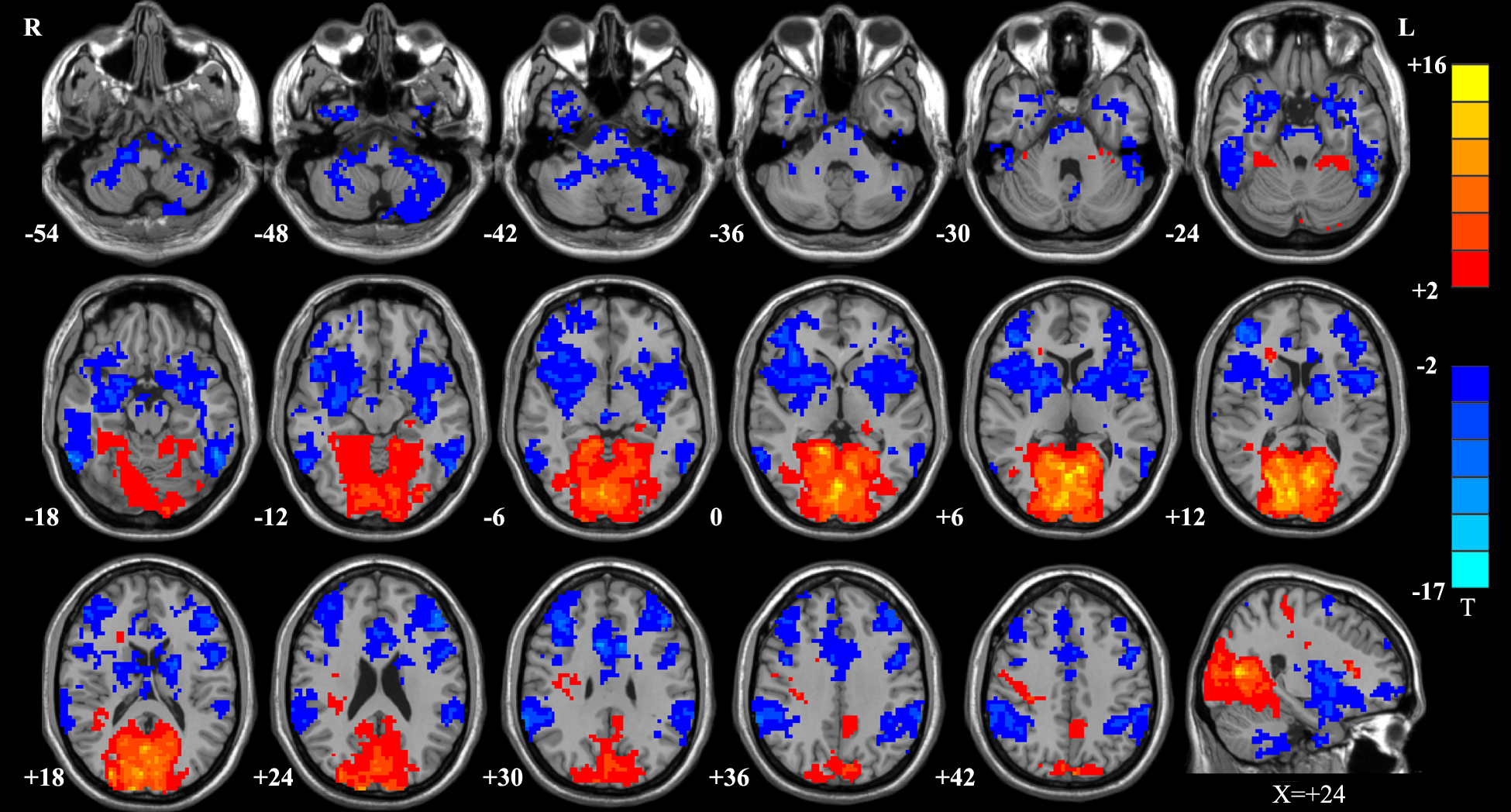

Supplement: S3 Fig — The warm color areas represent the regions that had positive connectivity with BA17, including the bilateral postcentral gyrus, the bilateral inferior part of precuneus, the bilateral posterior cingulate gyrus, the Subcortical structure of right frontal lobe, and the bilateral occipital lobe. In contrast, the cool color areas showed the regions negatively connected with BA17, which were located at the bilateral superior/middle/inferior frontal gyrus, the bilateral anterior cingulate gyrus, the bilateral corpus callosum, the bilateral thalamus, the bilateral supramarginal gyrus, the bilateral superior parietal lobule, the bilateral parahippocampal gyrus, and the bilateral middle/inferior temporal gyrus. (TIF) [file pone.0139819.s003.tif]

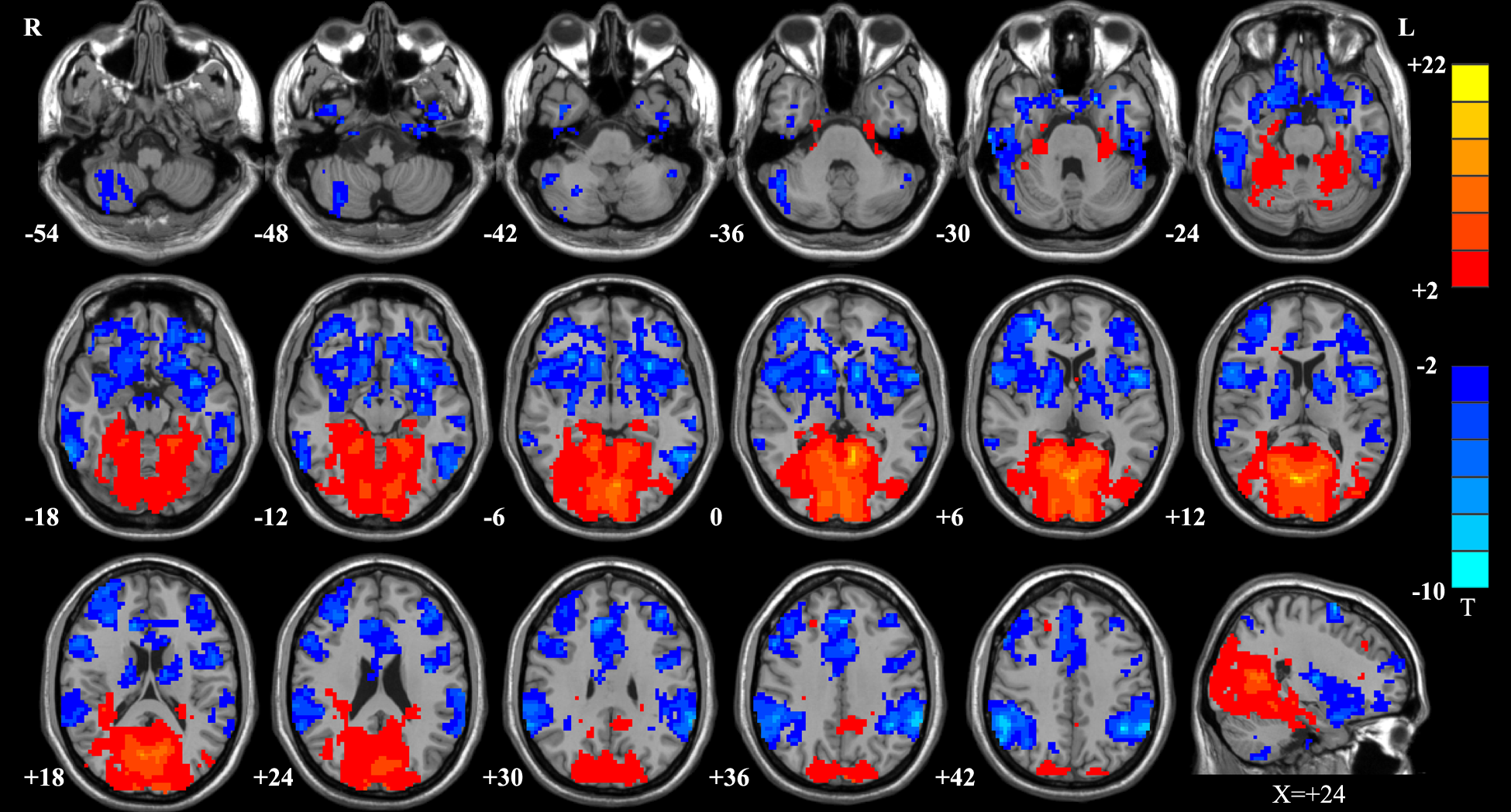

Supplement: S4 Fig — The warm color areas represented the regions that had positive connectivity with BA17, including the bilateral fusiform gyrus, the bilateral inferior part of precuneus, the bilateral posterior cingulate gyrus, the bilateral occipital lobe. In contrast, the cool color areas showed the regions negatively connected with BA17, which were located at the bilateral superior/middle/inferior frontal gyrus, the bilateral anterior cingulate gyrus, the bilateral middle/inferior temporal gyrus, the bilateral superior parietal lobule, the bilateral parahippocampal gyrus, the bilateral corpus callosum, the bilateral supramarginal gyrus, and the bilateral brain stem. (TIF) [file pone.0139819.s004.tif]
