# Supplementary material for: The Brain Activity in Brodmann Area 17: A Potential Bio-Marker to Predict Patient Responses to Antiepileptic Drugs
Source: PLoS One. 2015 Oct 6;10(10):e0139819. doi: 10.1371/journal.pone.0139819 (PMC4595505; doi:10.1371/journal.pone.0139819)
Supplement: S1 File — Table A. Seizure type and medication information for the patients. Table B. Regions showing fALFF differences between SUC patients and healthy controls. Table C. Regions showing fALFF differences between SUC patients and SC patients. Table D. Regions showing fALFF differences between DR patients and healthy controls. Table E. Regions showing fALFF differences between DR patients and WH patients. Table F. Regions showing fALFF differences between SC patients and healthy controls. Table G. Regions showing fALFF differences between WH patients and healthy controls. Table H. Overlap regions with common fALFF changes between SUC vs. CON and DR vs. CON. Table I. Overlap regions with common fALFF changes between SUC vs. WH and DR vs. SC. Table J. Significant differences in functional connectivity to the BA17 area among SUC, SC and healthy controls. Table K. Significant differences in functional connectivity to the BA17 area between SUC patients and healthy controls. Table L. Significant differences in functional connectivity to the BA17 area between SC patients and healthy controls. Table M. Significant differences in functional connectivity to the BA17 area between SUC and SC patients. Table N. Comparison of clinical features associated with early identification of patient’s response to AEDs between SUC and SC group. (DOC) [file pone.0139819.s005.doc]

**Supporting tables**

**Table A**. Seizure type and medication information for the patients

| **Number** | **Seizure type ^a^** | **AEDs before scanning** | **AEDs while scanning** | **AEDs after scanning** | **Group** |
| --- | --- | --- | --- | --- | --- |
| 1 | NO | NO | NO | NO | Control |
| 2 | NO | NO | NO | NO | Control |
| 3 | NO | NO | NO | NO | Control |
| 4 | NO | NO | NO | NO | Control |
| 5 | NO | NO | NO | NO | Control |
| 6 | NO | NO | NO | NO | Control |
| 7 | NO | NO | NO | NO | Control |
| 8 | NO | NO | NO | NO | Control |
| 9 | NO | NO | NO | NO | Control |
| 10 | NO | NO | NO | NO | Control |
| 11 | NO | NO | NO | NO | Control |
| 12 | NO | NO | NO | NO | Control |
| 13 | NO | NO | NO | NO | Control |
| 14 | P-GS | NO | NO | VPA | SUC |
| 15 | P-GS | NO | NO | VPA | SUC |
| 16 | P-GS | NO | NO | TPM, LTG | SUC |
| 17 | P-GS | NO | NO | VPA | SUC |
| 18 | P-GS | NO | NO | VPA, TPM | SUC |
| 19 | P-GS | NO | NO | TPM, LTG | SUC |
| 20 | P-GS | NO | NO | TPM | SUC |
| 21 | P-GS | NO | NO | VPA | SUC |
| 22 | P-GS | NO | NO | TPM | SUC |
| 23 | P-GS | NO | NO | TPM | SUC |
| 24 | P-GS | NO | NO | LTG | SUC |
| 25 | P-GS | NO | NO | LTG | SC |
| 26 | P-GS | NO | NO | VPA | SC |
| 27 | P-GS | NO | NO | VPA | SC |
| 28 | P-GS | NO | NO | TPM | SC |
| 29 | P-GS | NO | NO | VPA | SC |
| 30 | P-GS | NO | NO | VPA | SC |
| 31 | P-GS | NO | NO | TPM | SC |
| 32 | P-GS | NO | NO | TPM | SC |
| 33 | P-GS | NO | NO | VPA | SC |
| 34 | P-GS | NO | NO | VPA | SC |
| 35 | P-GS | CBZ, PB | CBZ+PB+TPM ^b^ | CBZ, PB, TPM | DR |
| 36 | P-GS | PB, CBZ | CBZ+VPA | CBZ, VPA, TPM, LEV | DR |
| 37 | P-GS | CBZ,PB,VPA | VPA | VPA,LTG,TPM | DR |
| 38 | P-GS | CBZ, PHT, VPA | VPA+TPM | VPA | DR |
| 39 | P-GS | PHT,PB,CBZ | CBZ | CBZ,VPA | DR |
| 40 | P-GS | CBZ, PHT, VPA | CBZ+TPM | CBZ, TPM | DR |
| 41 | P-GS | PHT, VPA, TPM, OXC | LTG+OXC | LTG, OXC | DR |
| 42 | P-GS | CBZ, TPM | TPM+VPA | TPM, VPA, OXC | DR |
| 43 | P-GS | VPA | VPA | VPA | WH |
| 44 | P-GS | VPA | VPA | VPA | WH |
| 45 | P-GS | PB,TPM | TPM | TPM | WH |
| 46 | P-GS | CBZ, VPA | TPM | TPM, OXC | WH |
| 47 | P-GS | CBZ,TPM | TPM | TPM | WH |
| 48 | P-GS | VPA,CBZ,TPM | CBZ+TPM | CBZ+TPM | WH |
| 49 | P-GS | PHT,CBZ,VPA | Withdraw ^c^ | Withdraw | WH |
| 50 | P-GS | VPA | Withdraw | Withdraw | WH |
| 51 | P-GS | VPA | Withdraw | Withdraw | WH |
| 52 | P-GS | CBZ | VPA | VPA | WH |
| 53 | P-GS | VPA | VPA | VPA | WH |

AEDs: anti-epileptic drugs, VPA: valproate, TPM: topiramate, LTG: lamotrigine, LEV: Levetiracetam, PB: phenobarbital, PHT: phenytoin, CBZ: carbamazepine, SUC: seizure-uncontrolled, SC: seizure-controlled, DR: Drug-resistant, WH: well-healed

^a^P-GS: all patients’ have the same seizure type of partial secondary generalized seizure,

^b^CBZ+PB+TPM: polytherapy by carbamazepine, phenbarbital and topiramate

^c^Withdraw: For some well-healed patients, physicians had made the decision to withdraw AED s

**Table B**. Regions showing fALFF differences between SUC patients and healthy controls

| **Brain regions ^a^** | **BA^b^** | **Voxels** | **T value** | **Hemisphere ^c^** | **X ^d^** | **Y** | **Z** |
| --- | --- | --- | --- | --- | --- | --- | --- |
| Left lingual gyrus | NA | 311 | 4.16 | L | -24 | -84 | -9 |
| Right lingual gyrus | NA | 46 | 4.67 | R | 18 | -78 | -12 |
| Left cuneus | NA | 41 | 5.20 | L | -9 | -78 | 12 |
| Right posterior cingulate | 30 | 31 | 4.42 | R | 15 | -66 | 9 |
| Left middle occipital gyrus | 39 | 30 | 4.08 | L | -39 | -78 | 9 |
| Right middle occipital gyrus | NA | 25 | 3.61 | R | 24 | -93 | 6 |
| Right cuneus | 17 | 21 | 5.28 | R | 6 | -99 | 6 |
| Right middle occipital gyrus | NA | 17 | 3.37 | R | 39 | -78 | 3 |
| Right inferior occipital gyrus | 19 | 18 | 3.16 | R | 36 | -81 | -12 |

**^a^**Significantly different brain regions.

**^b^**The Brodmann area where the peak voxel was located.

**^c^**The hemisphere where the peak voxel was located.

**^d^**The x, y, z, coordinates of the peak voxel in the Montreal Neurological Institute (MNI) space.

**Table C**. Regions showing fALFF differences between SUC patients and SC patients

| **Brain regions** | **BA** | **Voxels** | **T value** | **Hemisphere** | **X** | **Y** | **Z** |
| --- | --- | --- | --- | --- | --- | --- | --- |
| Left cuneus | 17 | 143 | 4.64 | L | -12 | -96 | 15 |
| Right cuneus | 17 | 63 | 3.99 | R | 12 | -102 | 9 |
| Left middle temporal-occipital area | 39 | 34 | 3.82 | L | -39 | -78 | 9 |
| Right fusiform gyrus | NA | 32 | 4.41 | R | 27 | -63 | -15 |
| Left lingual gyrus | NA | 30 | 3.23 | L | -9 | -69 | 9 |
| Right middle temporal-occipital area | NA | 27 | 3.41 | R | 51 | -75 | 6 |
| Right lingual gyrus | 17 | 20 | 3.49 | R | 18 | -93 | -15 |

**Table D**. Regions showing fALFF differences between DR patients and healthy controls

| **Brain regions** | **BA** | **Voxels** | **T value** | **Hemisphere** | **X** | **Y** | **Z** |
| --- | --- | --- | --- | --- | --- | --- | --- |
| Left middle occipital gyrus | 18 | 56 | 4.38 | L | -36 | -87 | 9 |
| Left cuneus | 17 | 50 | 4.43 | L | -15 | -93 | 3 |
| Right middle temporal-occipital area | NA | 49 | 4.38 | R | 27 | -75 | 12 |
| Right fusiform | 17 | 35 | 3.85 | R | 30 | -78 | -9 |
| Right cuneus | 17 | 31 | 4.10 | R | 18 | -102 | 12 |
| Right middle occipital gyrus | NA | 26 | 4.97 | R | 39 | -81 | 3 |
| Left fusiform | NA | 17 | 4.18 | L | -33 | -66 | -6 |

**Table E**. Regions showing fALFF differences between DR patients and WH patients

| **Brain regions** | **BA** | **Voxels** | **T value** | **Hemisphere** | **X** | **Y** | **Z** |
| --- | --- | --- | --- | --- | --- | --- | --- |
| Right fusiform | NA | 196 | 5.27 | R | 42 | -54 | -3 |
| Left fusiform | NA | 90 | 4.83 | L | -30 | -69 | -6 |
| Left cuneus | 17 | 62 | 3.63 | L | -18 | -99 | 6 |
| Right middle occipital gyrus | NA | 37 | 4.76 | R | 39 | -87 | 3 |

**Table F**. Regions showing fALFF differences between SC patients and healthy controls

| **Brain regions** | **BA** | **Voxels** | **T value** | **Hemisphere** | **X** | **Y** | **Z** |
| --- | --- | --- | --- | --- | --- | --- | --- |
| Left inferior occipital gyrus | NA | 37 | 3.69 | L | -45 | -75 | -15 |
| Right fusiform gyrus | NA | 20 | -3.92 | R | 30 | -63 | -9 |

**Table G**. Regions showing fALFF differences between WH patients and healthy controls

| **Brain regions** | **BA** | **Voxels** | **T value** | **Hemisphere** | **X** | **Y** | **Z** |
| --- | --- | --- | --- | --- | --- | --- | --- |
| Right fusiform gyrus | NA | 17 | -3.66 | R | 30 | -66 | -6 |

**Table H. Overlap regions with common fALFF changes between SUC vs. CON and** DR vs. CON

| **Brain regions** | **BA** | **Voxels** | **T value** | **Hemisphere** | **X** | **Y** | **Z** |
| --- | --- | --- | --- | --- | --- | --- | --- |
| Left cuneus, middle occipital gyrus | 17 | 41 | 13.69 | L | -15 | -93 | 3 |
| Left middle occipital gyrus | 39 | 27 | 13.17 | L | -39 | -78 | 9 |
| Right middle occipital gyrus | NA | 13 | 13.69 | R | 39 | -78 | 3 |
| Right superior occipital gyrus, middle occipital gyrus, cuneus | 19 | 6 | 8.79 | R | 21 | -99 | 9 |
| Right lingual gyrus | 17 | 5 | 9.1 | R | 18 | -84 | -12 |
| Right inferior occipital gyrus | 19 | 5 | 11.21 | R | 36 | -81 | -12 |
| Left fusiform, inferior occipital gyrus | NA | 4 | 8.33 | L | -33 | -60 | -9 |
| Subcortical structure of left occipital lobe, inferior occipital gyrus | NA | 3 | 7.01 | L | -30 | -72 | -6 |
| Left cuneus | 17 | 2 | 6.75 | L | -12 | -87 | 9 |
| Subcortical structure of right temporal lobe | NA | 2 | 12.17 | R | 30 | -63 | 12 |
| Right cuneus | 17 | 1 | 5.16 | R | 18 | -93 | 3 |
| Right middle occipital gyrus | NA | 1 | 6.37 | R | 48 | -78 | 0 |

**Table I. Overlap regions with common fALFF changes between SUC vs. WH and** DR vs. SC

| **Brain regions** | **BA** | **Voxels** | **T value** | **Hemisphere** | **X** | **Y** | **Z** |
| --- | --- | --- | --- | --- | --- | --- | --- |
| Right superior occipital gyrus, cuneus, middle occipital gyrus | 19 | 27 | 10.8 | R | 21 | -99 | 9 |
| Left fusiform, lingual gyrus | 19 | 14 | 9.6 | L | -21 | -81 | -18 |
| Right cerebellum posterior lobe, fusiform | NA | 14 | 9.11 | R | 30 | -63 | -18 |
| Left middle occipital gyrus | NA | 12 | 10.98 | L | -42 | -78 | 6 |
| Left middle occipital gyrus | NA | 11 | 9.69 | L | -33 | -93 | 3 |
| Right middle occipital gyrus | NA | 10 | 8.6 | R | 42 | -81 | 3 |
| Right fusiform, lingual gyrus | 18 | 5 | 7.44 | R | 24 | -78 | -15 |
| Left middle occipital gyrus | 18 | 2 | 6.8 | L | -18 | -99 | 3 |

**Table J. Significant differences in functional connectivity to the BA17 area among SUC, SC and healthy controls**

| **Brain region ^a^** | **BA** | **T value** | **X** | **Y** | **Z** |
| --- | --- | --- | --- | --- | --- |
| Left precuneus | 30 | 4.43 | -3 | -54 | 15 |
| Right precuneus | NA | 7.89 | 12 | -63 | 24 |
| Left posterior cingulate | NA | 5.79 | -6 | -42 | 33 |
| Right posterior cingulate | NA | 6.72 | 9 | -42 | 30 |
| Left middle cingulate | NA | 5.21 | -6 | -36 | 33 |
| Right middle cingulate | NA | 10.69 | 6 | 33 | 33 |
| Left anterior cingulate | NA | 12.56 | -9 | 30 | 27 |
| Right anterior cingulate | NA | 7.17 | 9 | 42 | 21 |
| Left middle frontal gyrus | NA | 5.4 | -24 | 30 | 36 |
| Right middle frontal gyrus | NA | 6.72 | 24 | 27 | 42 |
| Left superior frontal gyrus | NA | 17.87 | -12 | 15 | 42 |
| Right superior frontal gyrus | NA | 13.63 | 21 | 33 | 36 |
| Left medial superior frontal gyrus | 32 | 10.77 | -3 | 21 | 39 |
| Left inferior parietal lobe | NA | 3.61 | -30 | -45 | 48 |
| Right inferior parietal lobe | NA | 6.39 | 30 | -48 | 51 |
| Left superior parietal lobule | NA | 18.99 | -18 | -63 | 60 |
| Right superior parietal lobule | NA | 12.67 | 21 | -66 | 48 |
| Left inferior occipital gyrus | NA | 4.46 | -24 | -84 | -9 |
| Left middle occipital gyrus | NA | 9.04 | -33 | -90 | 9 |
| Right middle occipital gyrus | NA | 8.33 | 42 | -81 | 6 |
| Left superior occipital gyrus | NA | 6.24 | -27 | -93 | 24 |
| Right superior occipital gyrus | NA | 5.9 | 27 | -84 | 27 |
| Right middle temporal gyrus | NA | 10.53 | 42 | -66 | 15 |
| Left fusiform | NA | 6.96 | -21 | -75 | -6 |
| Right fusiform | 20 | 9.86 | 30 | -3 | -42 |
| Left midbrain | NA | 13.1 | -3 | -15 | -9 |
| Right midbrain | NA | 8.42 | 3 | -18 | -9 |
| Right parahippocampal gyrus | NA | 9.86 | 30 | -3 | -42 |
| Left thalamus | NA | 9.36 | -9 | -18 | 12 |
| Right thalamus | NA | 8.17 | 6 | -12 | 12 |
| Left lingual gyrus | 18 | 4.94 | -12 | -78 | -12 |
| Right lingual gyrus | NA | 4.11 | 15 | -57 | 0 |
| Subcortical structure of right frontal lobe | NA | 8.99 | 30 | 27 | 15 |

**^a^**Brain region where the peak voxel was located

**Table K**. Significant differences in functional connectivity to the BA17 area between SUC patients and healthy controls

| **Brain region** | **BA** | **Voxels** | **One sample t test results**^a^ | | **T value** | **Connectivity change**^b^ | **MNI coordinates** | | |
| --- | --- | --- | --- | --- | --- | --- | --- | --- | --- |
|  |  |  | **SUC** | **Control** |  |  | **X** | **Y** | **Z** |
| Inferior part of precuneus, posterior cingulate | NA | 1104 | Negative | Positive | -4.88 | Negative change | 0 | -15 | -9 |
| Superior frontal gyrus, anterior cingulate，middle frontal gyrus | NA | 677 | Negative | Negative | -5.46 | Negative increased change | 9 | 24 | 18 |
| Midbrain | NA | 39 | Negative | Negative | -3.76 | Negative increased change | 9 | -30 | -6 |
| Left superior parietal lobule | NA | 253 | Positive | None | 5.95 | Positive change | -18 | -63 | 60 |
| Right superior parietal lobule | 7 | 141 | Positive | None | 4.66 | Positive change | 21 | -63 | 51 |
| Right superior occipital gyrus, middle occipital gyrus | NA | 104 | Positive | Positive | 3.16 | Positive increased change | 21 | -81 | 18 |
| Right middle temporal gyrus | NA | 67 | Positive | Negative | 4.17 | Positive change | 33 | 3 | -42 |
| Left superior occipital gyrus, middle occipital gyrus, | 19 | 55 | Positive | Positive | 3.77 | Positive increased change | -27 | -90 | 27 |
| Left middle occipital gyrus | 19 | 44 | Positive | Positive | 3.01 | Positive increased change | -24 | -87 | 6 |

^a^ One sample t test: The significantly positive connectivity, the significantly negative connectivity and no significant connectivity between the ROI and other parts of the brain for each group were defined by one-sample t-test. And here we present the connectivity style for voxels based on one sample t test results..

^b^ Connectivity change means comparing with the control group how the connectivity of SUC group changes. If the types of connectivity of the two groups are the same based on the one sample t test result, we used the descriptions of negative increased change or positive increased change. If the types of connectivity of the two groups are different, we used negative change or positive change to point the direction of connectivity changes of the SUC group.

**Table L**. Significant differences in functional connectivity to the BA17 area between SC patients and healthy controls

| **Brain region** | **BA** | **Voxels** | **One sample t test results** | | **T value** | **Connectivity change** ^a^ | **MNI coordinates** | | |
| --- | --- | --- | --- | --- | --- | --- | --- | --- | --- |
|  |  |  | **SC** | **Control** |  |  | **X** | **Y** | **Z** |
| Left middle occipital gyrus | 19 | 154 | Positive | Positive | -5.75 | Positive decreased change | -45 | -84 | 12 |
| Right middle occipital gyrus | 19 | 54 | Positive | Positive | -3.92 | Positive decreased change | 48 | -81 | 9 |
| Right middle temporal gyrus | NA | 35 | Negative | Negative | -3.64 | Negative increased change | 42 | -66 | 15 |
| Right fusiform | 36 | 24 | Negative | Positive | -3.63 | Negative change | 24 | -6 | -48 |
| Right parahippocampal gyrus | NA | 22 | Negative | Negative | -3.30 | Negative increased change | 24 | -15 | -27 |
| Right lingual gyrus | NA | 23 | Positive | Positive | -2.86 | Positive decreased change | 15 | -51 | 0 |
| Subcortical structure of right frontal lobe | NA | 28 | Positive | Negative | 4.63 | Positive change | 27 | 24 | 15 |

^a^ Connectivity change means comparing with the control group how the connectivity of SC group changes. If the types of connectivity of the two groups are the same based on the one sample t test result, we used the descriptions of positive decreased change and negative increased change. If the types of connectivity of the two groups are different, we used negative change or positive change to point the direction of connectivity changes of the SC group.

**Table M**. Significant differences in functional connectivity to the BA17 area between SUC and SC patients

| **Brain region** | **BA** | **Voxels** | **One sample t test results** | | **T value** | **Connectivity change** ^a^ | **MNI coordinates** | | |
| --- | --- | --- | --- | --- | --- | --- | --- | --- | --- |
|  |  |  | **SUC** | **SC** |  |  | **X** | **Y** | **Z** |
| Anterior cingulate, superior frontal gyrus, middle frontal gyrus | NA | 668 | Negative | Negative | -7.06 | Negative increased change | -12 | 15 | 42 |
| Inferior part of precuneus, posterior cingulate | 31 | 464 | Negative | Positive | -4.35 | Negative change | -3 | -36 | 42 |
| Right thalamus | NA | 351 | Negative | Negative | -4.78 | Negative increased change | 6 | -6 | 6 |
| Inferior part of precuneus, posterior cingulate | NA | 57 | Negative | Positive | -3.66 | Negative change | -15 | -60 | 18 |
| Left superior occipital gyrus, left middle occipital gyrus | NA | 412 | Positive | Positive | 5.99 | Positive increased change | -21 | -87 | 24 |
| Right middle occipital gyrus | NA | 225 | Positive | Positive | 6.43 | Positive increased change | 18 | -99 | 21 |
| Right fusiform, right parahippocampal gyrus | 36 | 158 | Positive | Negative | 5.36 | Positive change | 27 | -3 | -39 |
| Left superior parietal lobule | NA | 132 | Positive | None | 4.82 | Positive change | -18 | -78 | 54 |
| Right superior parietal lobule | NA | 89 | Positive | None | 4.77 | Positive change | 21 | -66 | 48 |
| Right superior occipital gyrus | NA | 30 | Positive | Positive | 4.20 | Positive increased change | 21 | -87 | 27 |

^a^ Connectivity change means comparing with the SC group how the connectivity of SUC group changes. If the types of connectivity of the two groups are the same based on the one sample t test result, we used the description of positive decreased change. If the types of connectivity of the two groups are different, we used negative change or positive change to point the direction of connectivity changes of the SUC group.

**Table N**. Comparison of clinical features associated with early identification of patient’s response to AEDs between SUC and SC group

| Clinical features | SUC | SC | P value |
| --- | --- | --- | --- |
| Age of first onset (years) | 23.91±9.41 | 29.70±17.20 | 0.34^a^ |
| Duration of disease (years) | 6.67±10.09 | 4.12±5.27 | 0.48 ^a^ |
| History of febrile convulsions (n, %) | 1(0.09) | 1(0.10) | 0.94^b^ |
| Number of seizures at baseline^c^ (onsets per month) | 6.67±7.12 | 1.87±2.77 | 0.06 ^a^ |
| Duration from last onset to fMRI scanning (hours) | 113.27±97.60 | 100.80±49.38 | 0.72 ^a^ |

^a^The P values were calculated by independent two-sample t tests

^b^The P value was calculated by Pearson Chi-square test

^c^Defined as the average number of seizures one month before fMRI scanning
